# ESM1 enhances fatty acid synthesis and vascular mimicry in ovarian cancer by utilizing the PKM2-dependent warburg effect within the hypoxic tumor microenvironment

**DOI:** 10.1186/s12943-024-02009-8

**Published:** 2024-05-08

**Authors:** Juan Zhang, Fan Ouyang, Anbo Gao, Tian Zeng, Ming Li, Hui Li, Wenchao Zhou, Qing Gao, Xing Tang, Qunfeng Zhang, Xiaomin Ran, Gang Tian, Xiyun Quan, Zhenzi Tang, Juan Zou, Yifei Zeng, Yunzhu Long, Yukun Li

**Affiliations:** 1 Department of Assisted Reproductive Centre, Zhuzhou Central Hospital, Xiangya Hospital Zhuzhou Central South University, Central South University, Zhuzhou, Hunan China; 2 Department of Cardiology, Zhuzhou Central Hospital, Xiangya Hospital Zhuzhou Central South University, Central South University, Zhuzhou, Hunan China; 3 Clinical Research Institute, The Second Affiliated Hospital, Hengyang Medical School, University of South China, Hengyang, Hunan China; 4 Hunan Province Key Laboratory of Tumor Cellular & Molecular Pathology, Cancer Research Institute, Hengyang Medical School, University of South China, Hengyang, Hunan China; 5 Trauma Center, Zhuzhou Central Hospital, Xiangya Hospital Zhuzhou Central South University, Central South University, Zhuzhou, Hunan China; 6 Department of Gynecologic Oncology, Hunan Cancer Hospital, The Affiliated Cancer Hospital of Xiangya School of Medicine, Central South University, Changsha, Hunan China; 7 Department of Rehabilitation, Zhuzhou Central Hospital, Xiangya Hospital Zhuzhou Central South University, Central South University, Zhuzhou, Hunan China; 8 Department of Pathology, Zhuzhou Central Hospital, Xiangya Hospital Zhuzhou Central South University, Central South University, Zhuzhou, Hunan China; 9 Department of Oncology, Shenzhen Luohu People’s Hospital, Shenzhen, Guangdong China; 10 Department of Infectious Disease, Zhuzhou Central Hospital, Xiangya Hospital Zhuzhou Central South University, Central South University, Zhuzhou, Hunan China

**Keywords:** Ovarian cancer, ESM1, PKM2, SUMOylation, Tumor metabolic reprogramming, Vascular mimicry

## Abstract

**Background:**

The hypoxic tumor microenvironment is a key factor that promotes metabolic reprogramming and vascular mimicry (VM) in ovarian cancer (OC) patients. ESM1, a secreted protein, plays an important role in promoting proliferation and angiogenesis in OC. However, the role of ESM1 in metabolic reprogramming and VM in the hypoxic microenvironment in OC patients has not been determined.

**Methods:**

Liquid chromatography coupled with tandem MS was used to analyze CAOV3 and OV90 cells. Interactions between ESM1, PKM2, UBA2, and SUMO1 were detected by GST pull-down, Co-IP, and molecular docking. The effects of the ESM1-PKM2 axis on cell glucose metabolism were analyzed based on an ECAR experiment. The biological effects of the signaling axis on OC cells were detected by tubule formation, transwell assay, RT‒PCR, Western blot, immunofluorescence, and in vivo xenograft tumor experiments.

**Results:**

Our findings demonstrated that hypoxia induces the upregulation of ESM1 expression through the transcription of HIF-1α. ESM1 serves as a crucial mediator of the interaction between PKM2 and UBA2, facilitating the SUMOylation of PKM2 and the subsequent formation of PKM2 dimers. This process promotes the Warburg effect and facilitates the nuclear translocation of PKM2, ultimately leading to the phosphorylation of STAT3. These molecular events contribute to the promotion of ovarian cancer glycolysis and vasculogenic mimicry. Furthermore, our study revealed that Shikonin effectively inhibits the molecular interaction between ESM1 and PKM2, consequently preventing the formation of PKM2 dimers and thereby inhibiting ovarian cancer glycolysis, fatty acid synthesis and vasculogenic mimicry.

**Conclusion:**

Our findings demonstrated that hypoxia increases ESM1 expression through the transcriptional regulation of HIF-1α to induce dimerization via PKM2 SUMOylation, which promotes the OC Warburg effect and VM.

**Supplementary Information:**

The online version contains supplementary material available at 10.1186/s12943-024-02009-8.

## Introduction

The ovaries require an extremely high volume of blood which is supplied directly by the abdominal aorta, in turn, ovarian cancer (OC) is dependent on nutrients and oxygen for its development and progression [1]. Most tumor microenvironments are hypoxic because of the high consumption of energy and oxygen [2–4]. Hypoxia induces epithelial-mesenchymal transition (EMT) and remodeling of glucose and lipid metabolism in cancer cells through a series of molecular signaling pathways to promote angiogenesis and vascular mimicry (VM), allowing them to adapt to the hypoxic microenvironment [5–8]. HIF-1α is a key protein in the hypoxic microenvironment that reshapes tumor metabolism and induces angiogenesis and vascular mimicry during the progression of multiple cancers [9, 10]. HIF-1α is directly involved in the adaptation of cancer cells from an oxidative phosphorylation state to an aerobic glycolysis state [11].

Endothelial cell specific molecule 1 (ESM1), also known as endocan, is a secreted protein that is involved in endothelium-dependent pathological disorders [12]. In our previous study, we found that ESM1 has an oncogenic function in OC development and progression by affecting angiogenesis [13]. According to Laloglu et al. [15], the serum concentration of ESM1 was found to be significantly elevated in patients with OC. Additionally, El Behery and colleagues suggested that ESM1 could serve as a valuable biomarker for predicting the prognosis of OC patients [14]. However, how ESM1 expression promotes OC progression, and its role in OC metabolic reprogramming is unclear.

During glycolysis, pyruvate kinase catalyzes the final step by converting phosphoenolpyruvate (PEP) into pyruvate [15]. Among the four isomers of mammalian pyruvate kinase, pyruvate kinase isozyme M2 (PKM2) is the predominant subtype in the majority of tissues [16]. PKM2 is highly expressed in a variety of tumor types, and it has been demonstrated to be an important diagnostic and prognostic marker for OC [17–19]. PKM2 exists in three distinct forms: an inactive monomer, a dimer with reduced activity, and a tetramer with full activity. The tetramer PKM2 is responsible for the rapid production of ATP through oxidative phosphorylation. On the other hand, the dimer PKM2 facilitates the Warburg effect by diverting glucose-derived carbon toward macromolecular biosynthesis but also assumes a nonglycolytic function within the nucleus, where it acts as a protein kinase to influence the gene transcription [20].

In this study, we found that ESM1 is a direct downstream target of HIF-1α and that ESM1 is responsible for OC cells vascular mimicry under hypoxic conditions. We found that a high level of ESM1 was involved in vascular mimicry, fatty acid synthesis, and the Warburg effect in OC through increased PKM2 SUMOylation. Finally, our study revealed the molecular mechanism of ESM1-derived STAT5 phosphorylation in OC cells.

## Methods

### Cell culture, transfection, and cobalt chloride treatment

CAOV3, OV90, A2780, SKOV3, and 293T cells were purchased from the American Type Culture Collection (ATCC; Manassas, VA, USA). These cells were cultured in McCoy’s 5a (SKOV3), DMEM (293T, CAOV3, and OV90), or RPMI 1640 (A2780) containing 10% (v/v) fetal bovine serum (FBS; Gibco, Invitrogen, Carlsbad, CA, USA) and 1% penicillin/streptomycin (GIBCO, CA, USA). Human ESM1-overexpressing lentiviruses were generated using GV341 vectors, while lentiviral-based small hairpin RNA (shRNA) targeting ESM1 (Targeting sequence: GCATCTGGAGATGGCAATATT) was constructed using GV112 vectors from HonorGene in Changsha, China. shRNAs targeting HIF-1α (Targeting sequence: GTGATGAAAGAATTACCGAAT) and PKM2 (Targeting sequence: GTTCGGAGGTTTGATGAAATC) were synthesized and purified by HonorGene in Changsha, China. Additionally, SUMO-specific protease 1 (SENP1), SUMO1, and PKM2 were cloned and inserted into the pcDNA3.1(+) vector with modifications, including pcDNA3.1(+)-MCS-6xHis, pcDNA3.1(+)-MCS-Myc, and pcDNA3.1(+)-MCS-GST. PKM2 point mutations were generated by HonorGene in Changsha, China.

Cobalt chloride (CoCl_2_) was used to replicate hypoxic conditions. The cells were initially seeded in dishes or plates and allowed to grow for 24 h in a complete medium. Subsequently, the medium was removed, and the cells were rinsed with PBS and incubated with 150 µM CoCl_2_ for 48 h.

### Collection of patient samples

Paraffin sections corresponding to tissue samples from a total of 30 patients were collected from Zhuzhou Central Hospital (Zhuzhou, Hunan, China), the Second Affiliated Hospital of the University of South China (Hengyang, Hunan, China), and the Affiliated Cancer Hospital of Xiangya School of Medicine (Changsha, Hunan, China) from 2020 to 2023, following the ethics standards of the Helsinki Declaration. None of the patients underwent radiotherapy or chemotherapy. Protocols were approved under Zhuzhou Central Hospital IRB protocols #ZZCHEC2023029-02.

### Bioinformatic analysis

The TCGA database (https://portal.gdc.cancer.gov) was accessed to retrieve and arrange the TCGA-OV (ovarian serous cystadenocarcinoma) project STAR process RNAseq data, as well as extract the data and clinical data in the TPM format. Subsequently, a Spearman analysis was conducted to examine the correlation between the variables, and the results were visualized using ggplot2 (version3.3.6).

### Luciferase reporter assay

293T cells were co-transfected with plasmids containing wild-type or mutant fragments from PKM2 and HIF-1α using Lipofectamine 3000. Luciferase activity was assessed utilizing the dual luciferase reporter assay system from Promega following a 48-hour incubation period. ESM1-Pro wt: ACTCATAAACGTGTAGGCAGA; ESM1-Pro mut: ACTCATTCCTCATAAGGCAGA.

### Western blot

Cells were collected using a drop of RIPA lysis buffer (R0020, Solarbio) supplemented with 1% 1 mM PMSF, and protein concentration was determined using a BCA protein quantification kit (SK1070, Solarbio). Normalized protein samples were added to a sampling buffer (P1040, Solarbio) and incubated at 100 °C for 5 min. Protein samples were loaded onto a 10% SDS gel for electrophoresis and transferred to a PVDF membrane. The PVDF membrane was blocked with 5% skim milk for 3 h at room temperature, incubated with primary antibody at 4 °C for 12 h, and washed with TBST three times for 10 min each. The membranes were then incubated with secondary antibody for 2 h at room temperature and washed 3 times with TBST for 5 min each. Finally, the PVDF membrane was analyzed for chemiluminescence development using Superkine West Femto Maximum Sensitivity Substrate (BMU102-CN, Abbkine). The primary antibodies used are as follows: ESM1 (Abcam, ab103590), STAT3 (Abcam, ab68153), p-STAT3 (Abcam, ab267373), VE-Cad (Abcam, ab205336), hypoxia-inducible Factor 1alpha (HIF-1α) (Abcam, ab51608), proliferating cell nuclear antigen (PCNA) (Abcam, ab265609), vimentin (Abcam, ab92547), PKM2 (CST # 4053), SUMO1 (CST#4930), SENP1 (CST#11,929), Flag (CST#14,793), Myc (CST#2276), GST (CST #2625), HA (CST#5017), His (CST#12,698), Histone H3 (CST#4499) and β-actin (Abcam, ab8226).

### Oil Red O staining

2.5 g of Oil Red O powder (AWI0457D, Abiowell) was dissolved into 50 ml of 100% isopropanol and subsequently diluted with distilled water at a ratio of 3:2 and filtered to obtain the oil Red O working solution. The cultured cells were washed with PBS, fixed with paraformaldehyde (AWI0056B, Abiowell) for 20 min, washed once with PBS, and treated with 60% isopropanol for 30 s. The cells were incubated with Oil Red O working solution for 15 min at room temperature in the dark. After three washes with PBS cells were stained with hematoxylin. Finally, the cells were washed with PBS and blocked using glycerol gelatin (AWI0180B, Abiowell).

### Immunohistochemistry (IHC)

An appropriate amount of endogenous peroxidase blocker (AFIHC001; AiFang Biological) was added dropwise to the tissue sections treated with citric acid repair solution (pH 6.0), and the mixture was incubated for 20 min at room temperature. The sections were then washed three times with PBST and the primary antibody was added dropwise to the tissue sections, which were incubated at 4 °C for 12 h. The sections were then washed three times with PBST for ten minutes each. An appropriate amount of secondary antibody (AFIHC001, AiFang Biological) was then added dropwise to the tissue sections, which were then incubated at room temperature for 20 min and washed 3 times with PBST for 5 min each. The tissue sections were color-developed using a 3,3’-diaminobenzidine (DAB) color development kit (AFIHC004, AiFang Biological). Finally, the sections were stained with hematoxylin and blocked with neutral resin.

### Immunofluorescence (IF)

Cells were fixed with paraformaldehyde (AWI0056B, Abiowell) for 30 min and incubated with 0.5% Triton X-100. The background was blocked BSA and cells were incubated with a primary antibody at 4 °C for 12 h followed by incubation with a fluorescent secondary antibody (AFIHC023; AiFang Biological) for 2 h at room temperature. After counterstaining with DAPI, the sections were observed under a confocal laser microscope (Leica TCS SP5II STED, Mannheim, Germany).

### RT‒PCR

RNAs were extracted from TRIzol (R401-01, Vazyme) and reversely transcribed according to the manufacturer’s instructions (R223, Vazyme). PCR was performed using the following primers (including ESM1-F: CCTTCGGGATGGATTGCAGA, R: CCGGCAGCATTCTCTTTCAC; Vimentin-F: GTCCGTGTCCTCGTCCTCCTAC, R: AGTTGGCGAAGCGGTCATTCAG; PKM-F: GGCTCGTGGTGATCTAGGCATTG, R. TGAGGTCTGTGGAGTGACTTGAGG; LDHA-F: CAGCCCGATTCCGTTACCTAATGG; R: CCTCATAAGCACTCTCAACCACCTG; HIF1α-F: ATCAGACACCTAGTCCTTCCGATGG, R. GTGGTAGTGGTGGCATTAGCAGTAG; β-actin-F: CCTGGCACCCAGCACAAT; R: GGGCCGGACTCGTCATAC).

### Coimmunoprecipitation (Co-IP) and mass spectrometry

Protein A/G agarose (Santa Cruz Biotechnology, USA) was used for immunoprecipitation (IP). Total proteins were extracted from the OC cell lines using IP lysis buffer, precleared with microspheres, and incubated with ESM1 antibodies at 4 °C overnight. The following day, the immune complex samples were incubated with protein A magnetic beads at room temperature for 20 min, and bound proteins were collected by centrifugation. The samples were diluted and boiled in SDS‒PAGE solution for subsequent Western blot analysis. The peptides were separated via SDS‒PAGE after dehydration with acetonitrile and subsequent drying with 100% acetonitrile. Subsequently, the extracted peptides were subjected to analysis using QExactive (QE) mass spectrometry (LC-MS/MS). Finally, KEGG enrichment analysis was employed to predict the molecular signaling pathways associated with the enriched proteins.

### Molecular docking

Molecular docking was performed as previously described [21]. The anticipated three-dimensional conformations of the underlying proteins ESM1, ubiquitin-like modifier-activating enzyme 2 (UBA2), and PKM2 were acquired from the AlphaFold protein database and the PDB database. Additionally, PyMOL software (version 4.3.0) was used to visualize the amino acid residues involved in interprotein interactions, while Explore Studio software was used to analyze the hydrogen bonding and hydrophobic interactions between the two proteins.

### Seahorse XF24 Extracellular acidification rate

A total of 1 × 10^5^ OC cells were plated in XF24-well plates. The glycolysis rate was measured using a Seahorse XF24 extracellular flux analyzer (Seahorse Bioscience). For real-time evaluation, Seahorse XF base medium was added to the XF24well plate, and glucose (10 mM), oligomycin (1 µM and 2 µM), and 2-deoxyglucose (2DG, 80 mM) were added in sequence according to the instructions.

### Tube formation assay

The 48-well plate was chilled at 4°C, and then 200 µl of pre-cooled matrix glue was added to each well, ensuring uniform coverage of the bottom. Afterward, the 48-well plate was incubated at 37 °C for 1 h. A total of 7.5 × 10^4^ cells were subsequently added to each well, and the plate was incubated for 6 h before microscopic evaluation.

### Transwell assay

Following the seeding of the indicated cells in suitable quantities into Transwell chambers (Costar, Cambridge, MA) fitted in 24-well culture plates, the plates were incubated for 16 h. Subsequently, the cultured cells were fixed using paraformaldehyde and stained with crystal violet to identify the cells that had successfully traversed or infiltrated the lower surface of the filter.

### Xenograft assay

A total of 5 × 10^6^ OC cells were injected subcutaneously into each group of BALB/c female nude mice (4–6 weeks). The subcutaneous tumor size was measured with a caliper every 12 days, and the tumor volume was calculated. The mice were euthanized on Day 48. The transplanted tumors were collected and weighed. The female nude mice in the Shikonin group were given Shikonin (1 mg/kg) by intraperitoneal injection once every 3 days, and the control group was given normal saline by intraperitoneal injection. All the above experiments were carried out in specific pathogen-free (SPF) animal laboratories. In addition, the animal experiments in this study were carried out following approval IRB protocol (#ZZCHEC2023029-02) and approved by the Ethics Committee of the Hospital Zhuzhou Central South University. All animal studies were reported according to the ARRIVE guidelines.

### Statistical analysis

All the results were analyzed by t-tests and one-way ANOVA with R language (version 3.6). Each assay included three biological replicates. A *p*-value < 0.05 was considered to indicate statistical significance.

## Results

### HIF-1α enhances endothelial cell specific molecule 1 (ESM1) expression to promote invasion and vascular mimicry in ovarian cancer cells in a hypoxic microenvironment in vitro

**Fig. 1:**
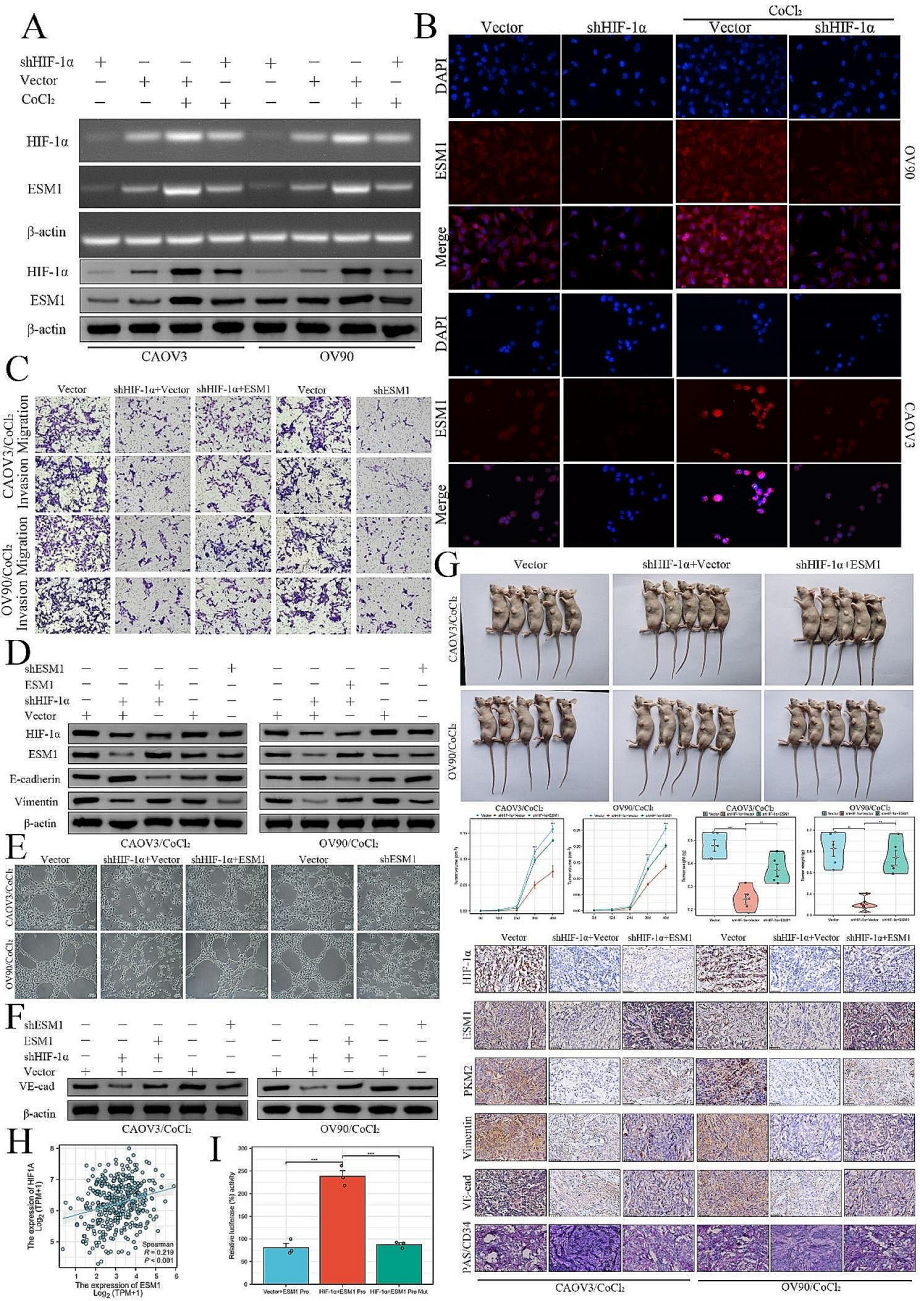
High HIF1-α upregulates ESM1 to promote OC cell migration and invasion and VM in a hypoxic microenvironment. (**A**) The mRNA and protein expression levels of HIF1-α and ESM1 in OC cells with CoCl_2_ or/and HIF1-α shRNA treatment were determined via Western blotting and PCR. (**B**) IF staining for the expression of ESM1 in OV90 and CAOV3 cells treated with CoCl_2_ or/and HIF1-α shRNA. (**C**) The migration and invasion ability of OV90/CoCl_2_ and CAOV3/CoCl_2_ cells transfected with HIF1-α shRNA, ESM1 shRNA, or ESM1 plasmid were evaluated via Transwell assays. (**D**) The expression of HIF1-α, ESM1, E-cadherin, and vimentin in OV90/CoCl_2_ and CAOV3/CoCl_2_ cells treated with HIF1-α shRNA, ESM1 shRNA or ESM1 plasmid was determined via Western blotting. (**E**) The VM ability of HIF1-α shRNA-, ESM1 shRNA- and ESM1 plasmid-treated OV90/CoCl2 and CAOV3/CoCl2 cells was evaluated via a tube formation assay. (**F**) The expression of VE-cadherin in OV90/CoCl_2_ and CAOV3/CoCl_2_ cells transfected with HIF1-α shRNA, ESM1 shRNA, or ESM1 plasmid was determined by Western blot analysis. (**G**) Xenograft models of OV90/CoCl_2_ and CAOV3/CoCl_2_ cells treated with HIF1-α shRNA and the ESM1 plasmid. Tumor growth curves, weights, and IHC staining for HIF1-α, ESM1, PKM2, vimentin, VE-cadherin, and PAS/CD34 are shown. (**H**) The correlation between HIF1-α and ESM1 was confirmed in the TCGA database OC dataset. (**I**) The binding ability of HIF1-α to the ESM1 promoter was confirmed by a dual-luciferase reporter gene assay in 293T cells. *P < 0.05, **P < 0.01, ***P < 0.001

To confirm the upregulation of ESM1 expression by HIF-1α in OC cells, we exposed genetically modified CAOV3 and OV90 cells expressing shRNA targeting HIF-1α to a 48-hour treatment with CoCl_2_ (200 µM), a hypoxia-mimicking agent. RT-PCR and Western blotting analyses demonstrated that hypoxia increases the expression of HIF-1α, while knockdown of HIF-1α led to the repression of ESM1 expression (Fig. 1A). Our IF results demonstrated that high HIF-1α levels induced by hypoxia upregulated ESM1 expression. Conversely, HIF-1α knockdown significantly repressed ESM1 expression (Fig. 1B).

In agreement with these findings, transwell assays demonstrated that restoring ESM1 expression effectively mitigated the inhibitory effects of HIF-1α knockdown on tumor cells migration and invasiveness. As expected, the knockdown of ESM1 efficiently counteracted the decreased migration and invasion of OC cells induced under hypoxic conditions (Fig. 1C). Western blot analysis demonstrated a reduction in Vimentin expression and an increase in E-cadherin expression upon HIF-1α knockdown, which was reversed by the overexpression of ESM1. The knockdown of ESM1 resulted in a decrease in Vimentin expression and an increase in E-cadherin expression (Fig. 1D).

VM is a highly patterned vascular channel formed by tumor cells. Previous studies have pointed out that lipid droplets play an important role in the spatial basis of VM [22]. Especially in the hypoxic microenvironment of OC cells, morpho-metabolic plasticity and VM are closely related [23]. In our recent study, we found that ESM1 promoted fatty acid synthesis in OC cells [21]. Therefore, we examined the effect of HIF-1α-ESM1 signaling axis on the expression of VE-cadherin, a key protein of VM, in OC cells. Tube formation assays showed that restoring ESM1 expression effectively mitigated the inhibitory effects of HIF-1α knockdown on VM while the knockdown of ESM1 counteracted the decrease in VM in OC cells induced by hypoxic conditions (Fig. 1E). Finally, Western blot analyses demonstrated a reduction in VE-cadherin expression upon HIF-1α knockdown, which was reversed by the overexpression of ESM1. Conversely, the knockdown of ESM1 resulted in a decrease in VE-cadherin expression (Fig. 1F).

### HIF-1α enhances ESM1 to accelerate ovarian cancer cells growth and vascular mimicry in a hypoxic microenvironment in vivo

To validate these findings in an in vivo setting, we used a xenograft model. When we subcutaneously injected OC cells with HIF-1α knockdown and/or ESM1 overexpression into nude mice, we observed fewer xenografts in the HIF-1α knockdown group compared to both the control group and the HIF-1α knockdown plus ESM1 overexpression group. However, the reintroduction of ESM1 effectively mitigated the inhibitory effects of HIF-1α knockdown on xenograft growth. In addition, the average sizes and weights of the xenografts derived from the HIF-1α knockdown group were significantly smaller than those from both the control group and the HIF-1α knockdown plus ESM1 overexpression group. Furthermore, IHC staining revealed significant decreases in the expression of PKM2, vimentin, and VE-cadherin in the HIF-1α knockdown group compared to both the control group and the HIF-1α knockdown plus ESM1 overexpression group. Finally, CD31/PAS double staining demonstrated a significant decrease in the level of VM in the HIF-1α knockdown group (Fig. 1G).

We then analyzed the correlation between HIF-1α and ESM1 in the TCGA database OC dataset, results from these studies indicated that HIF-1α was significantly and positively correlated with ESM1 in OC patients (Fig. 1H). The positive effect of HIF-1α on ESM1 transcriptional activity was further tested using a luciferase assay. Results from these studies demonstrated that compared with the vector control or ESM1 mutated vector, HIF-1α could significantly increase the transcriptional activity of ESM1 (Fig. 1I).

### Endothelial cell specific molecule 1 promotes the remodeling of glycolipid metabolism in ovarian cancer by driving the AMPK/mTOR pathway

**Fig. 2:**
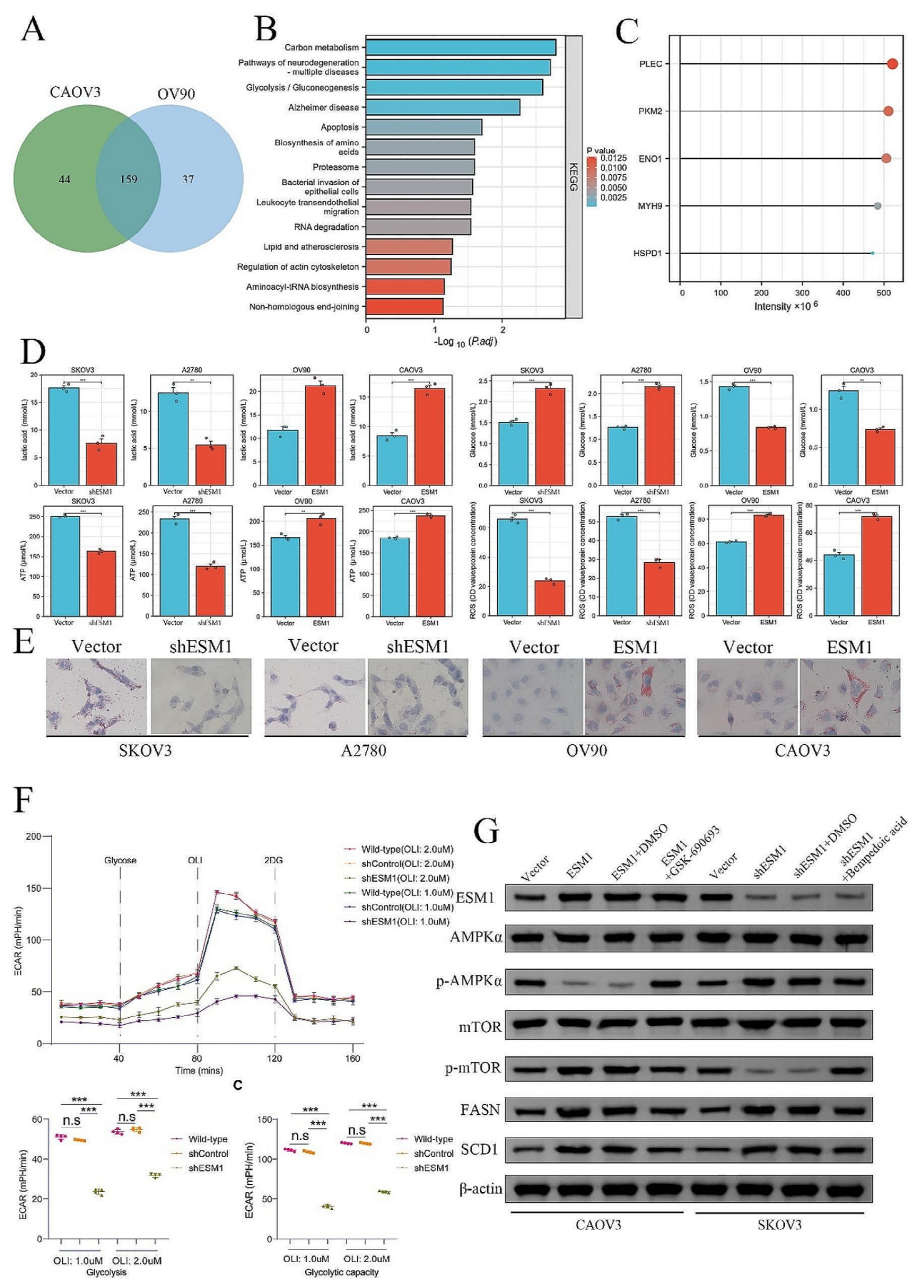
ESM1 promotes metabolic reprogramming by driving the ATP/AMPK/mTOR pathway in OC cells. (**A**) Venn diagram illustrating the proteins that interact with ESM1 in CAOV3 and OV90 cells. (**B**) KEGG enrichment results for these ESM1-binding proteins. (**C**) The top 5 proteins of these ESM1-binding proteins. (**D**) The effects of ESM1 on the levels of ROS, ATP, lactic acid, and glucose in SKOV3, A2780, OV90, and CAOV3 cells. (**E**) The effects of ESM1 on lipid metabolism in SKOV3, A2780, OV90, and CAOV3 cells were determined by Oil Red O staining. (**F**) The extent of extracellular acidification was confirmed in empty vector-treated and ESM1-knockdown OV90 cells. (**G**) The expression of ESM1, AMPK, p-AMPK, mTOR, p-mTOR, FASN, and SCD1 in CAOV3 and SKOV3 cells transfected with the ESM1 plasmid, ESM1 shRNA, GSK-690,693, or/and bempedoic acid. *P < 0.05, **P < 0.01, ***P < 0.001

To investigate the molecular mechanism underlying the involvement of ESM1 in the progression of OC, we performed IP and liquid chromatography coupled with tandem LC-MS/MS. ESM1-interacting protein complexes were isolated from CAOV3 and OV90 cells using anti-Flag monoclonal antibodies and paramagnetic beads. Through proteomic analysis, we successfully identified a total of 203 proteins as ESM1-specific binding proteins in CAOV3 cells and 196 proteins in OV90 cells. Interestingly, there was an overlap of 159 proteins that were found to interact with ESM1 in both cell lines (Fig. 2A). In addition, KEGG analysis revealed that these 159 ESM1-specific binding proteins were mostly enriched in carbon metabolism, glycolysis/gluconeogenesis, lipids, and atherosclerosis (Fig. 2B). The 5 proteins related to the strongest binding capacity of ESM1 were PLEC, PKM2, ENO1, MYH9, and HSPD1 (Fig. 2C). VM is a key molecular process in tumor evolution, which depends on metabolic reprogramming [24]. In particular, PKM2, a key enzyme in glucose metabolism, plays an important role in the formation of VM [25].

To provide additional evidence of the substantial role of ESM1 in glucose metabolism and lipid metabolism, we performed metabolic experiments to determine changes in glycolysis-related products. Additionally, we utilized Oil Red O staining to detect differences in the amount of intracellular lipids. We found that the levels of lactic acid and ATP were significantly greater in the ESM1-overexpressing group and lower in the ESM1-knockdown group when compared to the vector group. Similarly, when compared to the vector group, the glucose and ROS levels were significantly elevated in the ESM1 knockdown group and decreased in the ESM1 overexpression group (Fig. 2D). Oil red O staining showed that ESM1 promoted lipid production in OV90 and CAOV3 cells, and ESM1 knockdown inhibited lipid production (Fig. 2E). To investigate the impact of ESM1 on glycolysis, we assessed the extracellular acidification rate (ECAR), a reliable indicator of glycolysis rate and capacity induced by glucose, through the utilization of the extracellular flux method (Seahorse). Remarkably, the suppression of ESM1 expression substantially prevented the observed increase in the ECAR (Fig. 2F). Since the AMPK/mTOR pathway plays an important role in the energy and nutrient perception progression of various hypoxic cells [26], we tested its possible involvement. Our studies showed that the expression of AMPK/mTOR pathway-related proteins and that p-mTOR, FASN, and SCD1 were increased but that p-AMPK was decreased by ESM1 overexpression in CAOV3 cells; these effects could be rescued by an AMPK inhibitor (GSK-690,693). Conversely, ESM1 knockdown repressed p-mTOR, FASN, and SCD1 expression but promoted AMPK phosphorylation in SKOV3 cells, which was rescued by an AMPK activator (bempedoic acid) (Fig. 2G).

### Endothelial cell specific molecule 1 promotes vascular mimicry in ovarian cancer via the dimeric PKM2-dependent Warburg effect

**Fig. 3:**
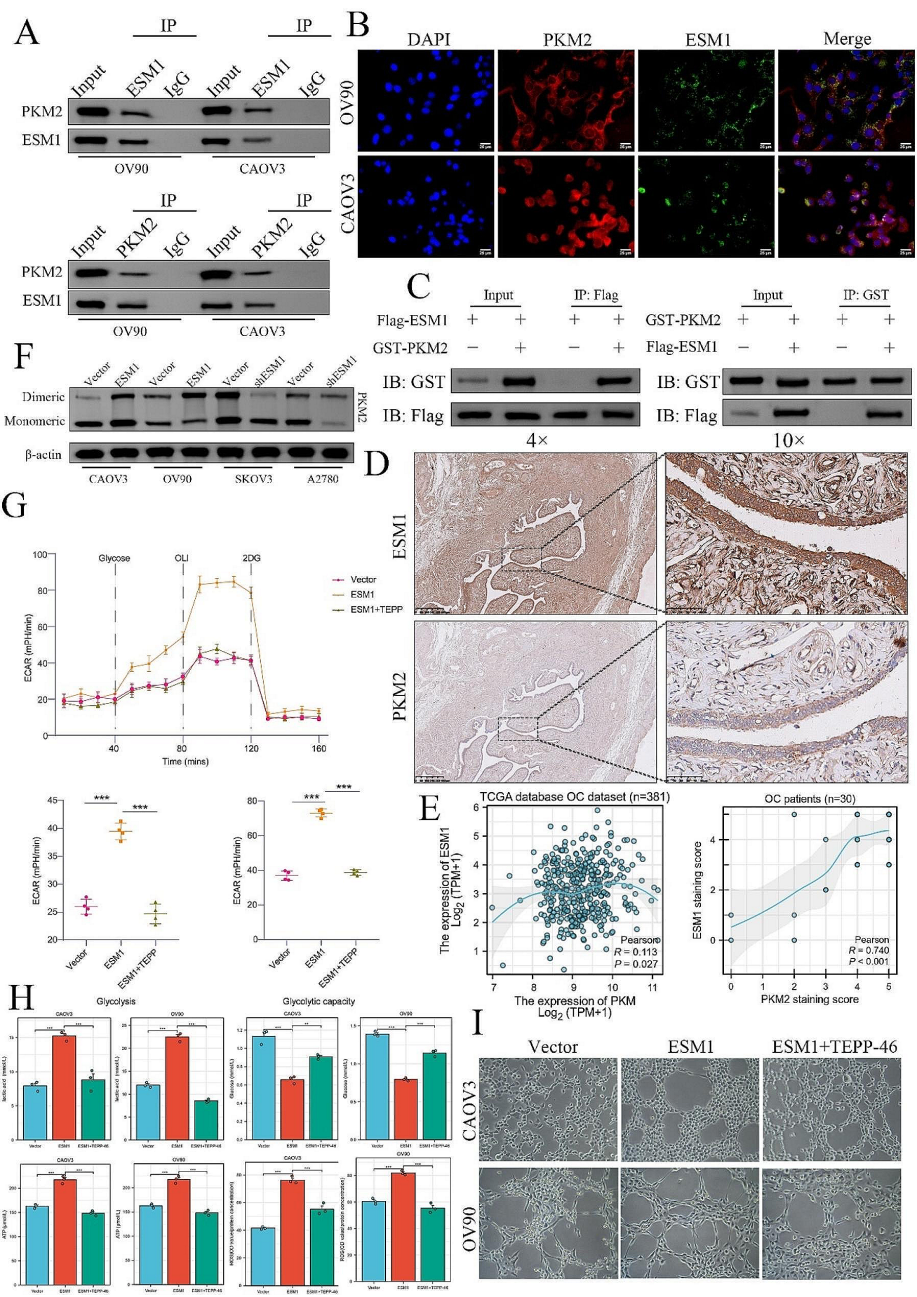
ESM1 drives the Warburg effect by interacting with PKM2 in OC cells. (**A**) A co-IP assay confirmed the interaction between ESM1 and PKM2 in CAOV3 and OV90 cells. (**B**) IF confirmed the interaction between ESM1 and PKM2 in CAOV3 and OV90 cells. (**C**) Flag-tagged ESM1 and GST-tagged PKM2 were transfected into HEK-293T cells, after which Co-IP was performed. (**D**) The expression of ESM1 and PKM2 in pathological serial sections of OC tissue samples determined by IHC staining (*n* = 30). (**E**) The correlation between ESM1 and PKM2 expression in OC patients is based on the TCGA database (*n* = 381). (**F**) The formation of dimeric PKM2 in OC cells with ESM1 knockdown or overexpression. (**G**) The extent of extracellular acidification was confirmed in OV90 cells transfected with vector, overexpressing ESM1 or overexpressing ESM1 plus TEPP. (**H**) The effects of ESM1 alone or in combination with TEPP on the levels of ROS, ATP, lactic acid, and glucose in OV90 and CAOV3 cells. (**I**) The ability of OV90 and CAOV3 cells to form VM via the vector, ESM1 overexpression, or ESM1 overexpression plus TEPP treatment, as determined by tube formation assay. *P < 0.05, **P < 0.01, ***P < 0.001

Among the proteins that interact with ESM1, PKM2 is a known key enzyme strongly associated with the Warburg effect (Fig. 2C). Our Co-IP analysis showed that endogenous ESM1 and PKM2 interact with each other in OV90 and CAOV3 cells (Fig. 3A). As expected, IF staining confirmed that ESM1 and PKM2 were strongly colocalized in these OC cells (Fig. 3B). Then, we overexpressed Flag-ESM1 and GST-PKM2 in **293T** cells and confirmed that exogenous ESM1 specifically binds to PKM2 in 293T transfected cells (Fig. 3C).

Next, we used IHC staining to investigate the expression and location of ESM1 and PKM2 in OC patient samples. These studies showed that ESM1 was significantly correlated with PKM2 (*P* < 0.001, *R* = 0.74) in OC patient samples (Fig. 3D). We also found that ESM1 mRNA was significantly correlated with PKM2 mRNA (*P* = 0.027, *R* = 0.113) in the TCGA database OC dataset (Fig. 3E).

According to a previous study, PKM2 dimers are believed to promote the development of various tumors through the Warburg effect. Hence, we hypothesized that ESM1 drives OC glycolysis to accelerate VM. Cross-linked PKM2 staining revealed that dimeric PKM2 was notably increased in the ESM1-overexpressing group compared to the vector group for CAOV3 and OV90 cells and was decreased in the ESM1-knockdown group compared to the vector group for SKOV3 and A2780 cells (Fig. 3F). TEPP-46, a PKM2 activator, has been shown to inhibit the dimerization of PKM2 [27]. Our studies using ECAR analysis revealed that TEPP-46 significantly restored the effect of ESM1 overexpression on the Warburg effect in OV90 cells (Fig. 3G). Moreover, we also found that TEPP-46 could restore the effects of ESM1 on lactic acid production, ATP production, ROS production, and glucose consumption (Fig. 3H). Tube formation assays also revealed that the effect of ESM1 on VM ability was significantly rescued by TEPP-46 (Fig. 3I). Taken together, these results suggested that the regulation of VM by ESM1 might involve the induction of the dimeric PKM2-dependent Warburg effect in OC.

### Endothelial cell specific molecule 1 mediates PKM2 SUMOylation and dimer formation via the UBA2-SUMO1 axis

**Fig. 4:**
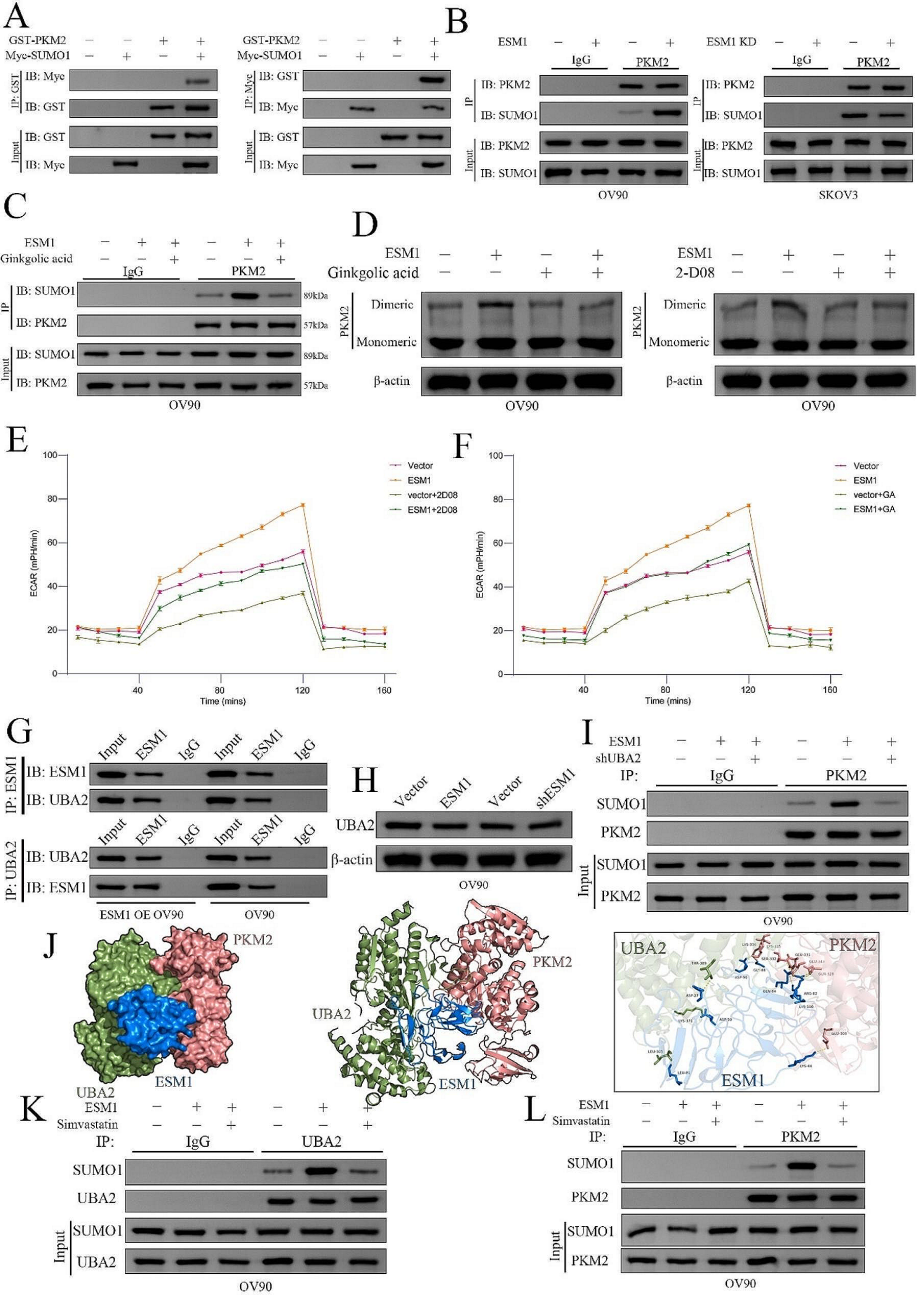
ESM1 promotes PKM2 SUMOylation to drive dimer formation via the UBA2/SUMO1 Axis (**A**) The interaction between SUMO1 and PKM2 was confirmed by IP and Western blotting in 293T cells. (**B**) The effects of ESM1 on the interaction between SUMO1 and PKM2 were confirmed by IP and Western blotting in OV90 and SKOV3 cells. (**C**) The effects of GA on ESM1-mediated promotion of PKM2 SUMOylation in OV90 cells. (**D**) The effects of GA or 2-D08 on dimeric PKM2 formation in OV90 cells. The extent of extracellular acidification was confirmed in OV90 cells transfected with vector, in cells overexpressing ESM1 plus 2-D08 (**E**), or in cells overexpressing ESM1 plus GA (**F**). (**G**) Co-IP assays confirmed the interaction between ESM1 and UBA2 in OV90 cells with or without ESM1 overexpression. (**H**) The effects of ESM1 on UBA2 expression in OV90 cells. (**I**) SUMOylation of PKM2 mediated by ESM1 was inhibited by UBA2 knockdown. (**J**) Molecular docking of UBA2, PKM2, and ESM1 by HADDOCK. Simvastatin inhibited the ESM1-induced interaction between SUMO1 and UBA2 (**K**) and the interaction between SUMO1 and PKM2 (**L**)

Previous studies have reported that SUMOylation is an important factor in the subcellular localization of the PKM2 protein and the performance of different molecular biological functions [28, 29]. However, the association between SUMOylation and dimeric PKM2 formation is still unclear. GSEA of ESM1 based on the TCGA database OC dataset showed that SUMOylation and glycolysis were significantly correlated with the molecular functions of ESM1 in OC patients (Supplementary Fig. 1). Hence, we hypothesized that ESM1 mediates PKM2 SUMOylation to promote PKM2 dimer formation in OC cells. To test this, we cotransfected GST-PKM2 and Myc-SUMO1 into HEK-293T cells and used Co-IP to demonstrate that PKM2 binds to SUMO1 (Fig. 4A). Moreover, we also found that ESM1 upregulates PKM2 protein SUMOylation in OV90 and SKOV3 cells (Fig. 4B). Ginkgolic acid (GA) and 2-D08 are small-molecule inhibitors of SUMOylation that can inhibit the SUMO-activating enzyme E1 and the SUMO E2 conjugating enzyme UBC9 [30, 31]. In our Co-IP experiments, GA was found to reverse the effects of ESM1 on the binding of the PKM2 protein to SUMO1 (Fig. 4C). Furthermore, Western blotting showed that GA and 2-D08 efficiently reversed the effects of ESM1 on PKM2 dimerization (Fig. 4D). Moreover, ECAR analysis revealed a positive effect of ESM1 on the Warburg effect in OV90 cells, which was restored by treatment with 2-D08 and GA (Fig. 4E&F). These results demonstrate that ESM1 facilitates the interaction of PKM2 with SUMO1 and increases SUMOylation to accelerate glycolysis.

UBA2 plays a crucial role in protein SUMOylation. UBA2 facilitates the attachment of SUMO1 to its C-terminus via a thioester bond with the cysteine residue in UBA2. Additionally, UBA2 activates SUMO1 by hydrolyzing ATP, resulting in the formation of a high-energy thioester bond [32]. Our LC-MS/MS analyses revealed that ESM1 binds to UBA2 in OV90 and CAOV3 cells (Fig. 2A). Therefore, we speculated that ESM1 could activate the SUMO1 protein through UBA2. Indeed, Co-IP revealed that exogenous and endogenous ESM1 and UBA2 bind to each other in OV90 cells (Fig. 4G). Moreover, Western blot analysis showed that ESM1 did not regulate the expression of UBA2 in OV90 cells (Fig. 4H).

To explore the possible involvement of UBA2 in facilitating ESM1-mediated PKM2 SUMOylation, we utilized shRNA to silence UBA2 expression. Subsequent Western blot analysis demonstrated that the inhibition of UBA2 hindered the enhancing effect of ESM1 on PKM2 SUMOylation (Fig. 4I).

Initially, the ESM1 molecular model was constructed using alphafold2. Subsequently, the molecular structure models of UBA2 (6XOG) and PKM2 (1ZJH) were obtained from the PDB database. GalaxyWEB docking software (https://galaxy.seoklab.org/c) was used for protein-protein docking, and ultimately, the optimal docking combination was selected through PyMOL mapping analysis. Eight residues, LEU81, ASP27, ASP50, ASP90, GLY88, ARG82, LYS100, and GLU303, in ESM1, were predicted to be crucial in the binding region. These data also indicated that the interaction among ESM1, PKM2, and UBA2 might be crucial for the molecular functions of ESM1 (Fig. 4J). The Western blot analysis revealed that the overexpression of ESM1 resulted in a notable activation of the SUMO1 protein. This activation was effectively suppressed by simvastatin, an inhibitor of ESM1 [29], through its interaction with UBA2 (Fig. 4K). Additionally, simvastatin was found to inhibit the PKM2 protein SUMOylation induced by ESM1 overexpression (Fig. 4L). Our findings indicate that ESM1 facilitates the activation of the SUMO1 through the UBA2, serving as a mediator that connects the activated SUMO1 protein with the PKM2 protein, thereby inducing PKM2 SUMOylation.

**Fig. 5:**
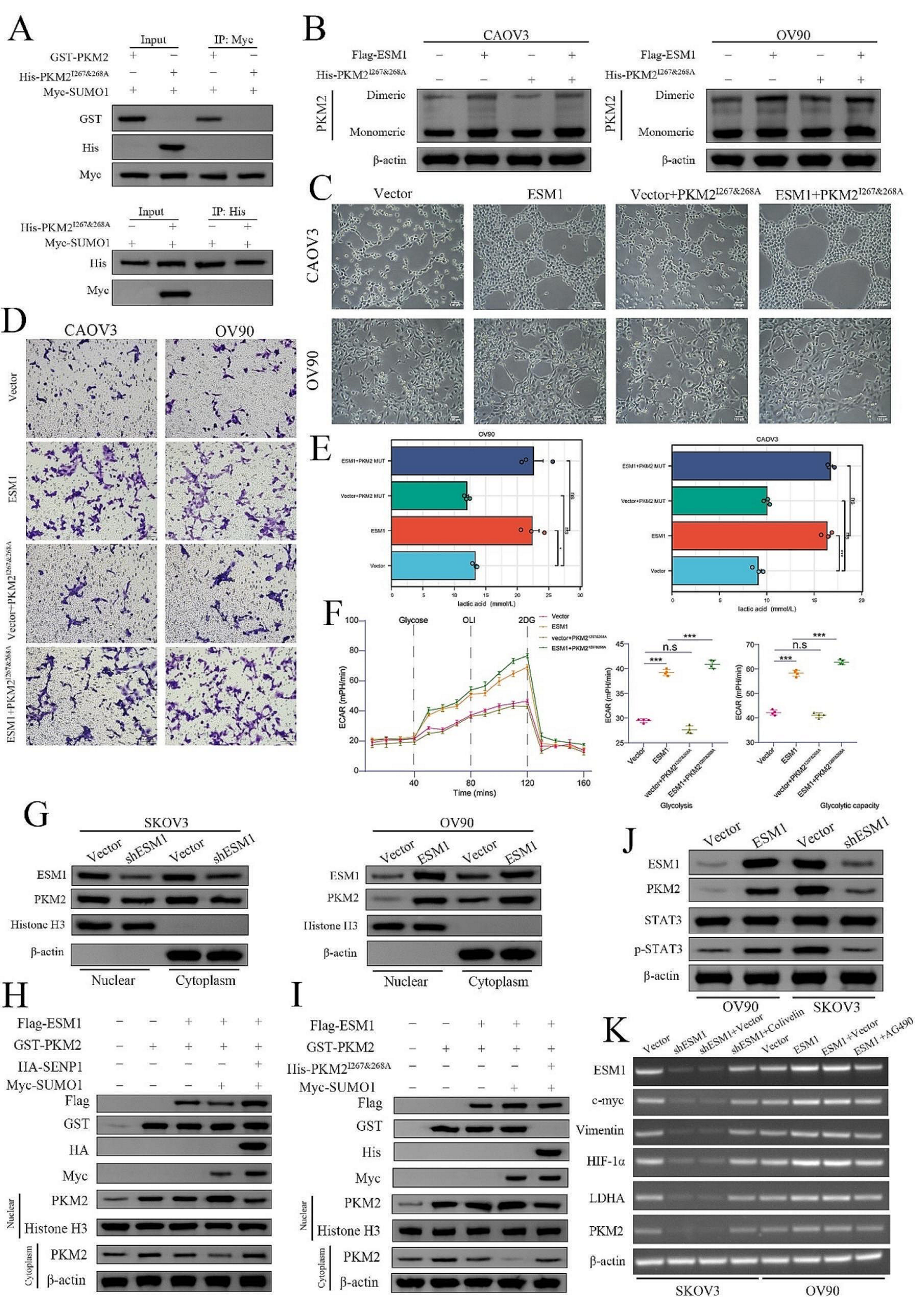
ESM1-induced PKM2 nuclear translocation is dependent on SUMOylation (**A**) The interaction between SUMO1 and PKM2 was confirmed by IP and Western blotting in 293T cells. (**B**) The effects of PKM2^I267&268 A^ on ESM1-induced dimeric PKM2 formation in CAOV3 and OV90 cells. (**C**) The VM ability of OV90 and CAOV3 cells transfected with vector, ESM1 overexpression vector, vector plus PKM2^I267&268 A,^ or ESM1 overexpression plus PKM2^I267&268 A^ was evaluated via a tube formation assay. (**D**) The invasion ability of OV90 and CAOV3 cells transfected with vector, ESM1 overexpression vector, vector plus PKM2^I267&268 A,^ or ESM1 overexpression plus PKM2^I267&268 A^ was evaluated via a Transwell invasion assay. (**E**) Lactic acid levels in OV90 and CAOV3 cells transfected with vector, ESM1 overexpression vector, vector plus PKM2^I267&268 A,^ or ESM1 overexpression plus PKM2^I267&268 A^. (**F**) The extent of extracellular acidification was confirmed in empty vector, ESM1-overexpressing vector, vector plus PKM2^I267&268 A,^ or ESM1-overexpressing plus PKM2^I267&268 A^ OV90 cells. (**G**) The effects of ESM1 on the cytoplasmic or nuclear distribution of PKM2 in SKOV3 and CAOV3 cells were determined via Western blotting. (**H**) The OV90 cell line was transfected with plasmids encoding Flag-tagged ESM1, GST-tagged PKM2, Myc-tagged SUMO1, and HA-tagged SENP1. Following transfection, a Western blot analysis was performed. (**I**) The OV90 cell line was transfected with plasmids encoding Flag-tagged ESM1, GST-tagged PKM2, Myc-tagged SUMO1, and HA-tagged PKM2^I267&268 A^. Following transfection, a Western blot analysis was performed. (**J**) The expression of ESM1, PKM2, STAT3, and p-STAT3 in OV90 and SKOV3 cells with ESM1 overexpression or ESM1 knockdown. K. The transcription levels of genes downstream of the STAT3 pathway in SKOV3 cells with ESM1 knockdown, ESM1 knockdown plus colivelin, OV90 cells with ESM1 overexpression, or ESM1 overexpression plus AG490. *P < 0.05, **P < 0.01, ***P < 0.001

Next, the SUMO moiety was identified by the SUMO-interacting motif (SIM) as the means for SUMOylation of a specific protein. A previous study demonstrated that the site at which SIM affects PKM2 is IKII_265 − 268_ and the introduction of point mutants at this SIM site, specifically PKM2^I267&268 A^, resulted in the complete elimination of SUMO1-induced SUMOylation of PKM2 [29]. We also confirmed that the SUMOylation site of PKM2 is IKII_265 − 268_ (Fig. 5A). Western blot analysis of ESM1-overexpressing CAOV3 and OV90 cells revealed that PKM2^I267&268 A^ could not form a dimer with PKM2 (Fig. 5B). Tube formation assay further showed that PKM2^I267&268 A^ did not promote VM capacity (Fig. 5C). Moreover, Transwell invasion assays also showed that, in contrast to wild-type PKM2, the invasion ability of CAOV3 and OV90 cells were no longer enhanced in the presence of PKM2^I267&268 A^ even though ESM1 was upregulated (Fig. 5D) and that PKM2^I267&268 A^ could not upregulate the level of lactic acid (Fig. 5E). ECAR analysis showed that PKM2^I267&268 A^ could not promote glycolysis (Fig. 5F). Collectively, these findings confirm that ESM1 plays a role in promoting the formation of dimeric PKM2 and facilitating the SUMOylation of PKM2 through the UBA2-mediated activation of SUMO1.

### Endothelial cell specific molecule 1 drives PKM2 nuclear translocation by regulating its SUMOylation

In aerobic glycolysis, the PKM2 dimer acts as a protein kinase in the nucleus to regulate the transcription of multiple genes and promote tumor progression [33]. Western blotting analyses showed that ESM1 knockdown impeded the nuclear translocation of PKM2 in SKOV3 cells, while ESM1 overexpression stimulates the nuclear translocation of PKM2 in CAOV3 cells (Fig. 5G). Moreover, the reduced PKM2 in both nuclear and cytoplasm upon shESM1 or increased PKM2 in both nuclear and cytoplasm upon ESM1 overexpression, which means ESM1 can regulate the expression of PKM2. In a previous study, SUMOylation was shown to play a significant role in PKM2 nuclear translocation [28, 34]. In conjunction with the findings, our hypothesis speculates that the regulation of PKM2 dimer formation by ESM1 involves SUMOylation and nuclear translocation, which occur dynamically and continuously. The enzyme SENP1 is responsible for deSUMOylation. To investigate the localization of PKM2 in OV90 cells, we conducted transfection experiments using plasmids expressing ESM1, PKM2, SUMO1, or SENP1. Western blot analysis revealed that ESM1-induced SUMOylation triggers the nuclear localization of the PKM2 protein, while SENP1 inhibits ESM1-mediated PKM2 nuclear localization through SUMOylation (Fig. 5H). Additionally, we conducted a transfection experiment in which OV90 cells were transfected with a plasmid expressing ESM1, PKM2, SUMO1, or PKM2^I267&268 A^. Subsequently, we examined the localization of PKM2. Western blot analysis revealed that co-transfection of OV90 cells with ESM1 and SUMO1 resulted in the nuclear translocation of PKM2. However, this effect was nullified in the presence of PKM2^I267&PKM2A^ (Fig. 5I). Previous research has demonstrated that nuclear PKM2 functions as a protein kinase, facilitating the phosphorylation of STAT3 at the tyrosine 705 residue [35]. Additionally, our investigation revealed that ESM1 plays a role in promoting the phosphorylation of STAT3 (Fig. 5J) and the transcription of downstream genes (Fig. 5K). These findings indicate that ESM1 governs both the expression of PKM2 and its translocation to the nucleus.

### The PKM2 inhibitor Shikonin attenuates progression of ovarian cancer and vascular mimicry

**Fig. 6:**
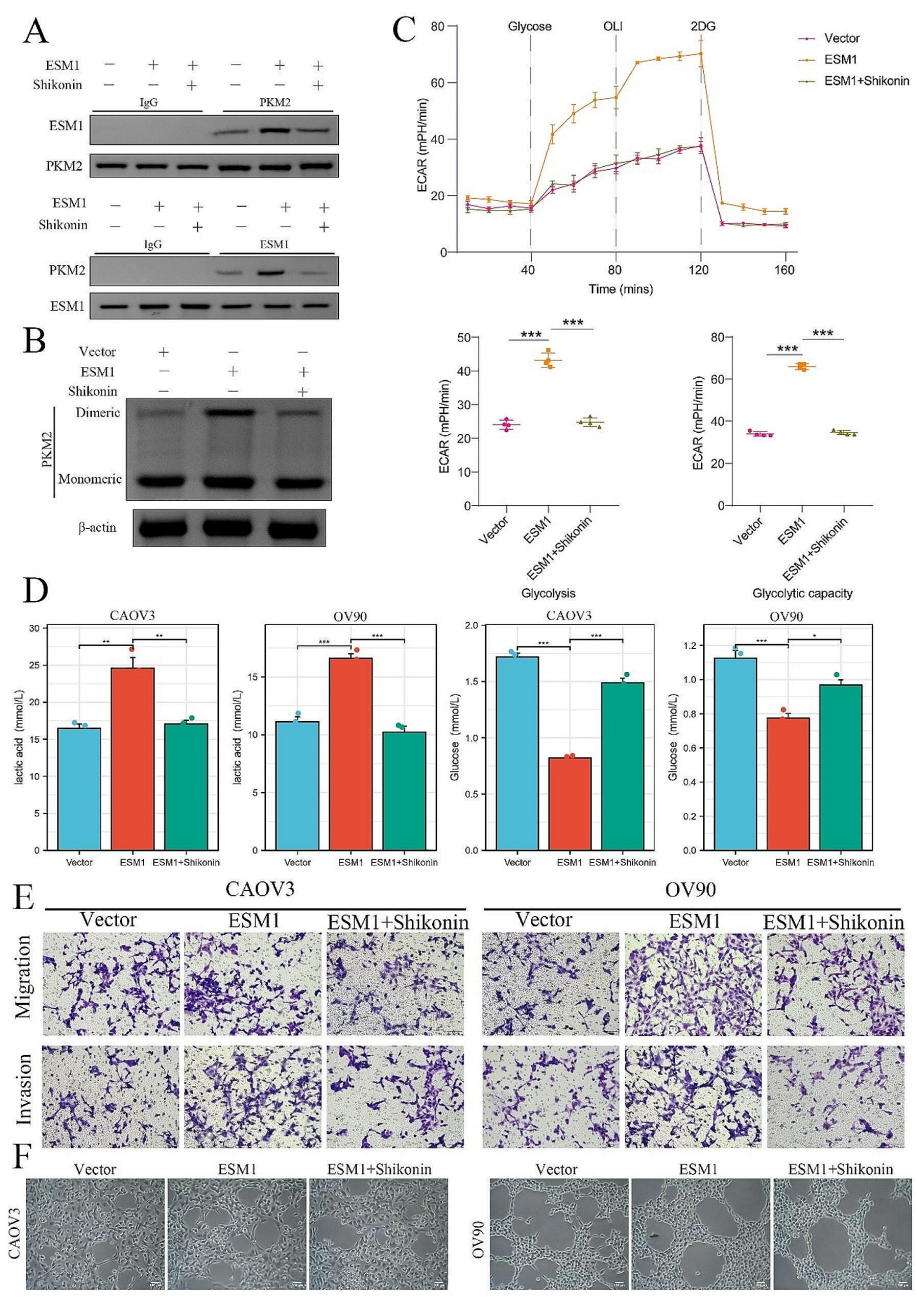
Shikonin represses OC cell VM and glycolysis by inhibiting the binding of ESM1 to PKM2 in vitro. (**A**) The effects of Shikonin on the interaction between ESM1 and PKM2 in OV90 cells overexpressing ESM1. (**B**) Western blot showing the formation of dimeric PKM2 in OV90 cells transfected with vector, ESM1, or ESM1 plus Shikonin. (**C**) The extent of extracellular acidification was confirmed in empty vector, ESM1-overexpressing, or ESM1-overexpressing plus Shikonin OV90 cells. (**D**) The levels of lactic acid and glucose in CAOV3 and OV90 cells treated with vector, ESM1, or ESM1 plus Shikonin. (**E**) The migration and invasion ability of OV90 and CAOV3 cells transfected with vector, with ESM1 overexpression, or with ESM1 overexpression plus Shikonin were evaluated via Transwell assays. (**F**) The ability of OV90 and CAOV3 cells to undergo VM via the vector, ESM1 overexpression, or ESM1 overexpression plus Shikonin treatment, as determined by a tube formation assay. *P < 0.05, **P < 0.01, ***P < 0.001

Shikonin is a specific PKM2 inhibitor [36–38]. Additionally, Shikonin has shown potent anticancer effects on a variety of tumors. However, the mechanisms underlying these effects are not fully understood. In the present study, we found that Shikonin attenuated the binding of ESM1 to PKM2 (Fig. 6A). Western blot analysis also revealed that Shikonin inhibited the expression of PKM2 in ESM1-overexpressing OV90 cells (Fig. 6B). Moreover, Shikonin inhibited glycolysis in OV90 cells (Fig. 6C). In both CAOV3 and OV90 cells, the levels of lactic acid were significantly reduced in the ESM1 and Shikonin group when compared to the ESM1-overexpressing group. Conversely, the glucose levels were significantly higher in the ESM1 and Shikonin group compared to the ESM1-overexpressing group (Fig. 6D). Functional experiments showed that Shikonin attenuated the effects of ESM1 on CAOV3 and OV90 cells migration, invasion, and VM capability (Fig. 6E&F). These results indicated that Shikonin could repress the interaction between PKM2 and ESM1 and the formation of PKM2 dimers to attenuate OC migration and invasion and VM by driving the Warburg effect in vitro.

**Fig. 7:**
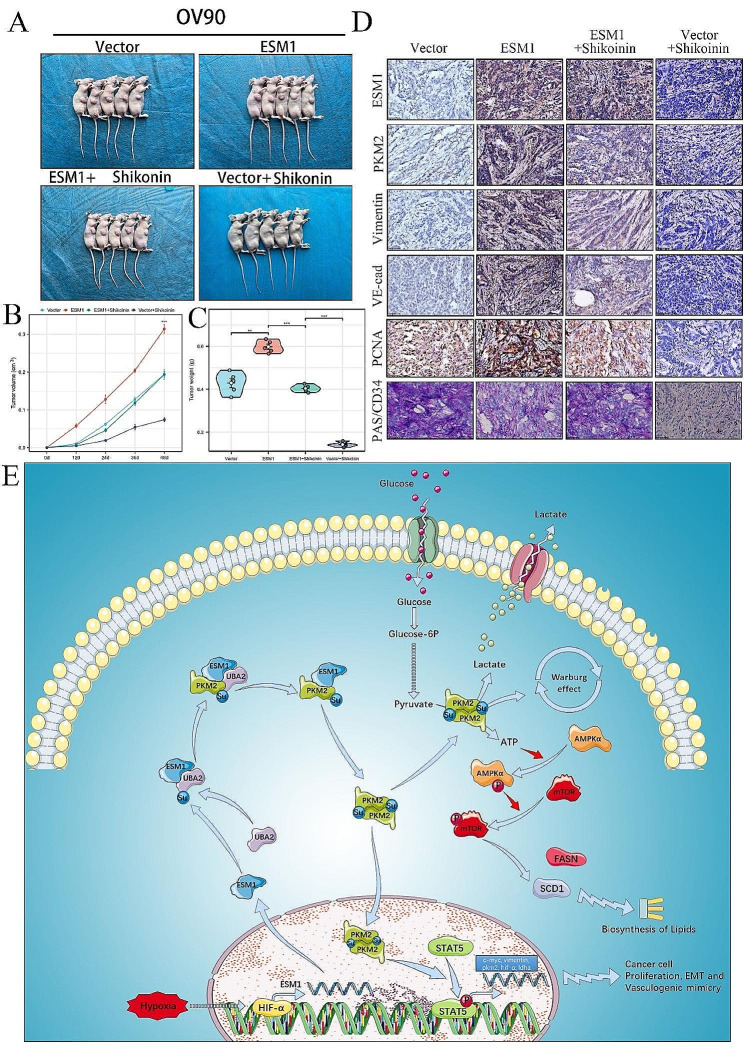
Shikonin represses OC cell growth, VM, and glycolysis in vivo. (**A**) Xenograft models of OV90 cells treated as indicated. Tumor growth curves (**B**) and tumor weights (**C**) in the xenograft models. (**D**) IHC staining for ESM1, PKM2, Vimentin, VE-cadherin, PCNA, and PAS/CD34 in these xenografts. (**E**) This study elucidates the molecular mechanism underlying the ESM1-PKM2 signaling axis in the hypoxic microenvironment of ovarian cancer. Hypoxia triggers the activation of HIF-1α, which subsequently upregulates ESM1 mRNA expression. ESM1, in turn, facilitates the activation of UBA2, leading to PKM2 SUMOylation. This process promotes the Warburg effect, resulting in increased ATP production, inhibition of AMPK phosphorylation, and promotion of mTOR phosphorylation. Furthermore, the activation of downstream FASN and SCD1 is induced, ultimately promoting fatty acid synthesis. Furthermore, the process of PKM2 SUMOylation enhances the nuclear localization of the dimer PKM2 and facilitates the phosphorylation of STAT3, thereby facilitating the transcription of downstream oncogenes to promote proliferation, EMT, and VM. These molecular mechanisms play pivotal roles in the progression of OC. *P < 0.05, **P < 0.01, ***P < 0.001

To further demonstrate the effects of ESM1 and Shikonin on OC growth and VM in vivo, we used OC xenograft tumor models. The results suggested that ESM1 can promote tumor growth, while Shikonin can inhibit the carcinogenic effect of ESM1 (Fig. 7A). Moreover, Shikonin effectively counteracted the effect of ESM1 on tumor volume and weight. Additionally, Shikonin itself significantly inhibited tumor growth (Fig. 7B&C). IHC staining revealed that the expression of PKM2, Vimentin, VE-cadherin, and PCNA was increased by ESM1 overexpression and that the presence of Shikonin significantly attenuated the effects of ESM1 (Fig. 7D). Moreover, VM was also enhanced by ESM1 overexpression but restored to normal levels upon treatment with Shikonin (Fig. 7D). Taken together, our results indicated that Shikonin significantly attenuates the OC growth and the VM of OC cells.

## Discussion

As a soluble sulfate skin proteoglycan, ESM1 is elevated in a variety of tumors and promotes progression of cancers, including proliferation, migration, invasion, angiogenesis, and drug resistance [12]. ESM1 is also an important member of tumor microenvironment and is absorbed by various cells, such as endothelial cells and neutrophils, and promotes immune escape and angiogenesis [39, 40]. In our previous studies, we found that ESM1 is a key factor in the occurrence and development of OC and can accelerate the proliferation, migration, invasion, escape of apoptosis, and angiogenesis of OC cells [13]. The mechanism is related to the activation of the PI3K-Akt signaling pathway [13, 41]. In addition, we also found that ESM1 and ANGPTL4 interact in OC cells, promoting angiogenesis and proliferation by influencing the remodeling of lipid metabolism and the activation of the STAT3 signaling pathway in OC and its microenvironment [21]. However, how ESM1 causes this series of kinase phosphorylation changes and metabolic remodeling is unknown.

The hypoxic microenvironment is one of the main characteristics of solid tumors and a major obstacle to cancer treatment [42]. Hypoxia has been extensively studied in tumor progression and is closely related to the Warburg effect and VM during tumorigenesis [3, 43]. However, the molecular mechanisms through which hypoxia regulates metabolism and VM remain unclear. OC is characterized by hypoxia and abnormal metabolic reprogramming concomitant with VM [44, 45]. Hypoxia-induced metabolic reprogramming and VM in OC have recently been reported [46, 47]. In this study, we found that hypoxia-induced the upregulation of HIF-1α expression, which can activate the transcription of ESM1.

In this study, we provided initial evidence regarding the potential mechanism through which hypoxia influences the transcriptional regulation of ESM1. Additionally, we confirmed the involvement of hypoxia in glycometabolic remodeling in OC and VM. However, there are likely other molecular mechanisms by which hypoxia regulates ESM1 that have not yet been verified, and further studies are needed to confirm how hypoxia regulates ESM1. Additionally, our study did not detect epigenetic factors involved in the regulation of ESM1 by the anoxic microenvironment, such as m6A modification and m5C modification. However, the role of the hypoxic tumor microenvironment in tumor cell epigenetic changes in tumor metabolic reprogramming and VM needs to be further explored.

In our study, we found that ESM1 regulates lipid remodeling by inducing the Warburg effect through PKM2 and regulating AMPK/mTOR signaling through ATP production. Previous studies have generally suggested that solid tumor cells use glycolysis to circumvent the TCA cycle or mitochondrial dysfunction for rapid productivity [48–50]. Additionally, cancer cells utilize VM, a biological process that mimics blood vessels and connects to normal vascular endothelial cells, to obtain additional nutrients and oxygen [51, 52]. Therefore, the Warburg effect and VM are two complementary molecular processes by which cancer cells respond to the anoxic microenvironment [24, 25, 53]. Through the application of IP mass spectrometry, we successfully identified PKM2 as the protein that interacts with ESM1. The formation of the PKM2 dimer is recognized as a crucial indicator of the Warburg effect [54]. Therefore, we investigated the regulatory role of ESM1 in PKM2 dimerization at the protein level and its effects on OC cells glycolysis. We demonstrated that ESM1 can interact with UBA2 and enhance SUMO1 activation and PKM2 SUMOylation. We hypothesize that ESM1 acts as a linker between the UBA2 protein and the PKM2 protein, bridging SUMO1 activation and inducing PKM2 SUMOylation. Subsequently, by analyzing ECAR and conducting lactic acid formation experiments, we observed that ESM1 stimulated the Warburg effect by promoting the formation of PKM2 in OC cells. Apart from its role in glucose metabolic remodeling, the PKM2 dimer also exhibits kinase activity, enabling it to phosphorylate downstream signaling pathways. Our results showed that in OC cells high ESM1 expression stimulates nuclear translocation of PKM2, promoting the phosphorylation of the STAT3 protein, and activating the transcription of downstream proto-oncogenes. This signaling pathway is also important for supporting the development of OC. Previous studies have shown that PKM2 SUMOylation can induce its nuclear translocation [29, 34]. In this study, we found that ESM1 can promote PKM2 nuclear translocation and the activation of the STAT3 signaling pathway by promoting PKM2 SUMOylation. In summary, our study revealed that the ESM1-PKM2 signaling axis plays a determinant role in promoting OC metabolic reprogramming and VM formation, one of the key mechanisms involved in the anoxic microenvironment (Fig. 7E). Additionally, Shikonin can inhibit OC growth through various mechanisms [55, 56]. We found that Shikonin can effectively inhibit the interaction between ESM1 and PKM2 and further inhibit the growth, glycolysis, and VM of orthotopic transplanted tumors.

## Conclusion

Our study revealed that ESM1 plays an essential role in promoting the Warburg effect and VM by facilitating the formation of dimers through PKM2 SUMOylation. Additionally, our findings demonstrated that Shikonin can impede the progression of OC by specifically inhibiting the interaction between ESM1 and PKM2, and can a promising candidate for OC treatment.

### Electronic supplementary material

Below is the link to the electronic supplementary material.

Supplementary Figure 1: GSEA for ESM1 based on the TCGA database OC dataset

## Data Availability

All the data generated or analyzed during this study are included in this published article.

## Abbreviations

VM: Vascular mimicry
OC: Ovarian cancer
ESM1: Endothelial cell specific molecule 1
PKM2: Pyruvate kinase isozyme M2
ECAR: Extracellular acidification rate
PEP: Phosphoenolpyruvate
shRNA: Small hairpin RNA
CoCl_2_: Cobalt chloride
IHC: Immunohistochemistry
IF: Immunofluorescence
Co-IP: Coimmunoprecipitation
MS: Mass spectrometry
IP: Immunoprecipitation
GA: Ginkgolic acid
UBA2: Ubiquitin-like modifier-activating enzyme 2
SENP1: SUMO-specific protease 1
PCNA: Proliferating cell nuclear antigen
