# Supplementary figures and images for: ESM1 enhances fatty acid synthesis and vascular mimicry in ovarian cancer by utilizing the PKM2-dependent warburg effect within the hypoxic tumor microenvironment
Source: Mol Cancer. 2024 May 8;23:94. doi: 10.1186/s12943-024-02009-8 (PMC11077861; doi:10.1186/s12943-024-02009-8)

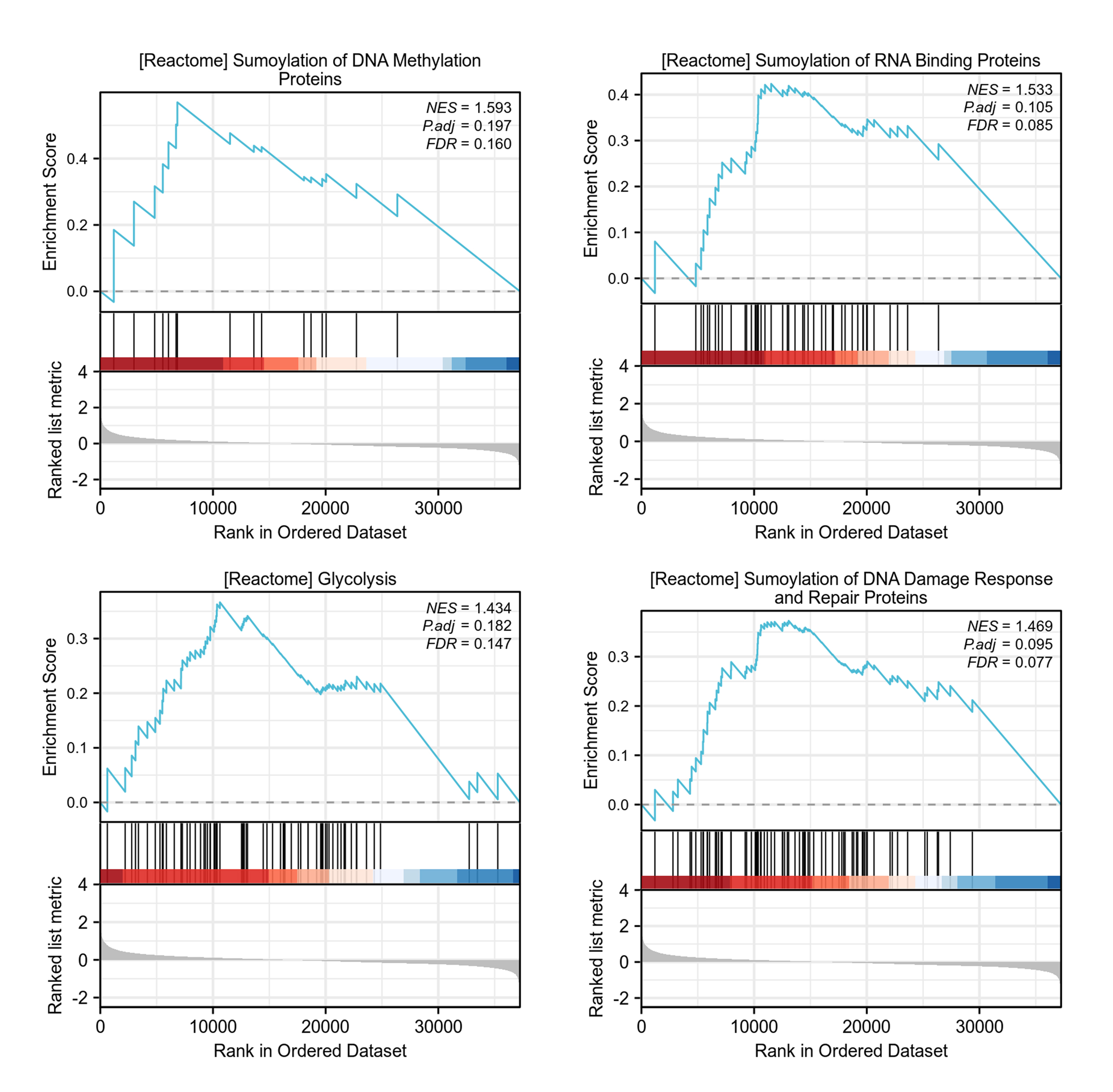

Supplement: Supplementary file 1 — Supplementary Figure 1: GSEA for ESM1 based on the TCGA database OC dataset [file 12943_2024_2009_MOESM1_ESM.jpg]
